# Development of a robust induced pluripotent stem cell atrial cardiomyocyte differentiation protocol to model atrial arrhythmia

**DOI:** 10.1186/s13287-023-03405-5

**Published:** 2023-07-27

**Authors:** Jordan Thorpe, Matthew D. Perry, Osvaldo Contreras, Emily Hurley, George Parker, Richard P. Harvey, Adam P. Hill, Jamie I. Vandenberg

**Affiliations:** 1 Victor Chang Cardiac Research Institute, Sydney, NSW Australia; 2 Department of Pharmacology, School of Biomedical Sciences, University of New South Wales, Sydney, Australia; 3 School of Clinical Medicine, Faculty of Medicine and Health, University of New South Wales, Sydney, Australia; 4 School of Biotechnology and Biomolecular Science, University of New South Wales, Sydney, NSW Australia

**Keywords:** Atrial cardiomyocyte, Stem cells, Cardiac, Arrhythmias, Atrial fibrillation

## Abstract

**Background:**

Atrial fibrillation is the most common arrhythmia syndrome and causes significant morbidity and mortality. Current therapeutics, however, have limited efficacy. Notably, many therapeutics shown to be efficacious in animal models have not proved effective in humans. Thus, there is a need for a drug screening platform based on human tissue. The aim of this study was to develop a robust protocol for generating atrial cardiomyocytes from human-induced pluripotent stem cells.

**Methods:**

A novel protocol for atrial differentiation, with optimized timing of retinoic acid during mesoderm formation, was compared to two previously published methods. Each differentiation method was assessed for successful formation of a contractile syncytium, electrical properties assayed by optical action potential recordings and multi-electrode array electrophysiology, and response to the G-protein-gated potassium channel activator, carbamylcholine. Atrial myocyte monolayers, derived using the new differentiation protocol, were further assessed for cardiomyocyte purity, gene expression, and the ability to form arrhythmic rotors in response to burst pacing.

**Results:**

Application of retinoic acid at day 1 of mesoderm formation resulted in a robust differentiation of atrial myocytes with contractile syncytium forming in 16/18 differentiations across two cell lines. Atrial-like myocytes produced have shortened action potentials and field potentials, when compared to standard application of retinoic acid at the cardiac mesoderm stage. Day 1 retinoic acid produced atrial cardiomyocytes are also carbamylcholine sensitive, indicative of active *I*_kach_ currents, which was distinct from ventricular myocytes and standard retinoic addition in matched differentiations. A current protocol utilizing reduced Activin A and BMP4 can produce atrial cardiomyocytes with equivalent functionality but with reduced robustness of differentiation; only 8/17 differentiations produced a contractile syncytium. The day 1 retinoic acid protocol was successfully applied to 6 iPSC lines (3 male and 3 female) without additional optimization or modification. Atrial myocytes produced could also generate syncytia with rapid conduction velocities, > 40 cm s^−1^, and form rotor style arrhythmia in response to burst pacing.

**Conclusions:**

This method combines an enhanced atrial-like phenotype with robustness of differentiation, which will facilitate further research in human atrial arrhythmia and myopathies, while being economically viable for larger anti-arrhythmic drug screens.

**Supplementary Information:**

The online version contains supplementary material available at 10.1186/s13287-023-03405-5.

## Background

Atrial fibrillation (AF) is the commonest clinically significant cardiac arrhythmia with a lifetime risk of approximately 1:3 [1, 2]. The incidence is also predicted to double in the next 40 years due to aging of the population and increasing incidence of metabolic syndrome [3]. Sustained AF is associated with serious complications including heart failure, stroke, and dementia [4–8]. AF also increases all-cause mortality, with approximately 5% of AF patients dying per year [9]. The recent SARS-COV-2 pandemic has also exacerbated AF risk by an additional 10.74 cases per 1000 persons, translating to an additional 44.05 cases per 1000 individuals who required hospitalization and 97.34 cases per 1000 patients requiring intensive care treatment [10].

The principal treatments for AF are catheter ablation or anti-arrhythmic drugs to control rate and/or rhythm. Ablation is only 50–80% effective long term [11, 12], and ~ 5% of procedures are associated with major complications [13]. Up to 70% of patients with persistent AF become refractory to anti-arrhythmic medication [14]. Understanding the mechanisms underlying AF and developing more effective and more affordable treatments are therefore an urgent healthcare need.

Most studies addressing the underlying mechanisms of atrial fibrillation have been undertaken in animal models. However, naturally occurring AF is almost nonexistent in most animal species, and significant differences in heart size, heart rate, and cellular electrophysiology have contributed to the poor translation of animal findings into new therapeutics [15]. Studies in human atrial samples have been invaluable; however, there is limited availability of this tissue compounded by the complex and heterogeneous nature of AF in humans. In recent years, there has been growing interest in developing human-induced pluripotent stem cell (hiPSC) models of AF [16–18]. HiPSCs can be utilized to create a potentially limitless source of cardiomyocytes, making them an ideal candidate for drug screening projects. HiPSCs generated from specific patients could also facilitate tailored therapy targeting specific disease mechanisms in those individuals.

Protocols to generate cardiomyocytes from hiPSCs primarily yield ventricular-like cardiomyocytes [19–22]. Addition of retinoic acid at the cardiac mesoderm stage favors production of atrial-like myocytes [23–26]. These cells show shortened action potentials, enhanced atrial-related gene expression profiles, and responsiveness to pharmacological inventions that primarily affect atrial myocytes. The yield and purity of atrial myocyte populations has been found to be improved by reducing BMP4 and Activin A concentration during mesoderm formation [27, 28]. One limitation of reducing BMP4 and Activin A, however, is that extensive re-optimization of growth factor concentrations is required for each cell line used [27, 28]. The increased cost, need for optimization of each line, as well as greater variability between differentiations of the same line, has limited the utilization of iPSC-derived atrial cardiomyocyte models for studying human disease.

Here, we investigated optimization of the timing of retinoic acid addition during cardiomyocyte differentiation with the aim of developing a robust and scalable protocol for generation of large amounts of atrial specific myocytes. Cardiac myocytes showed atrial-like electrophysiology in a total of 6 iPSC lines, derived from different individuals, tested without line specific optimization. Our robust protocol for production of iPSC-derived atrial cardiomyocytes should facilitate mechanistic studies into the molecular and cellular basis of AF and enable development of an anti-arrhythmic drug screening platform.

## Materials and methods

### Aim and design

This study aimed to find an optimal way of producing atrial cardiomyocytes derived from iPSCs; to this end, two existing methods from the literature were compared to a novel protocol described within. Utilizing two iPSC lines, 15C1 and 100C1, the three methods were compared to each other and the ventricular base protocol for: action potentials, field potential durations, and response to carbamylcholine, an *I*_kach_ activator. Further characterization was performed on the protocol described within, including an expanded set of iPSC lines, and the ability to form arrhythmic rotor activity.

### iPSC cell culture

hiPSC lines 15-C1 (16 y/o Male), 100-C1 (unknown aged Female), 8-C1 (unknown aged Male), 477-C1 (unknown aged Male), 273C1 (42 y/o Female) with no observed karyotypic abnormalities were generated by the Stanford Cardiovascular Institute Biobank from healthy patients as previously described [29]. Line VG1-C as characterized in Holliday et al. [30] was a generous gift from Christopher Semsarian, Centenary Institute. Cell lines were maintained with ReLeSR™ (05872, STEMCELL Technologies) using mTeSR Plus (100-0276, STEMCELL Technologies) on hESC pre-screened Matrigel (354277, Corning).

### Cardiomyocyte differentiation and dissociation

**Fig. 1:**
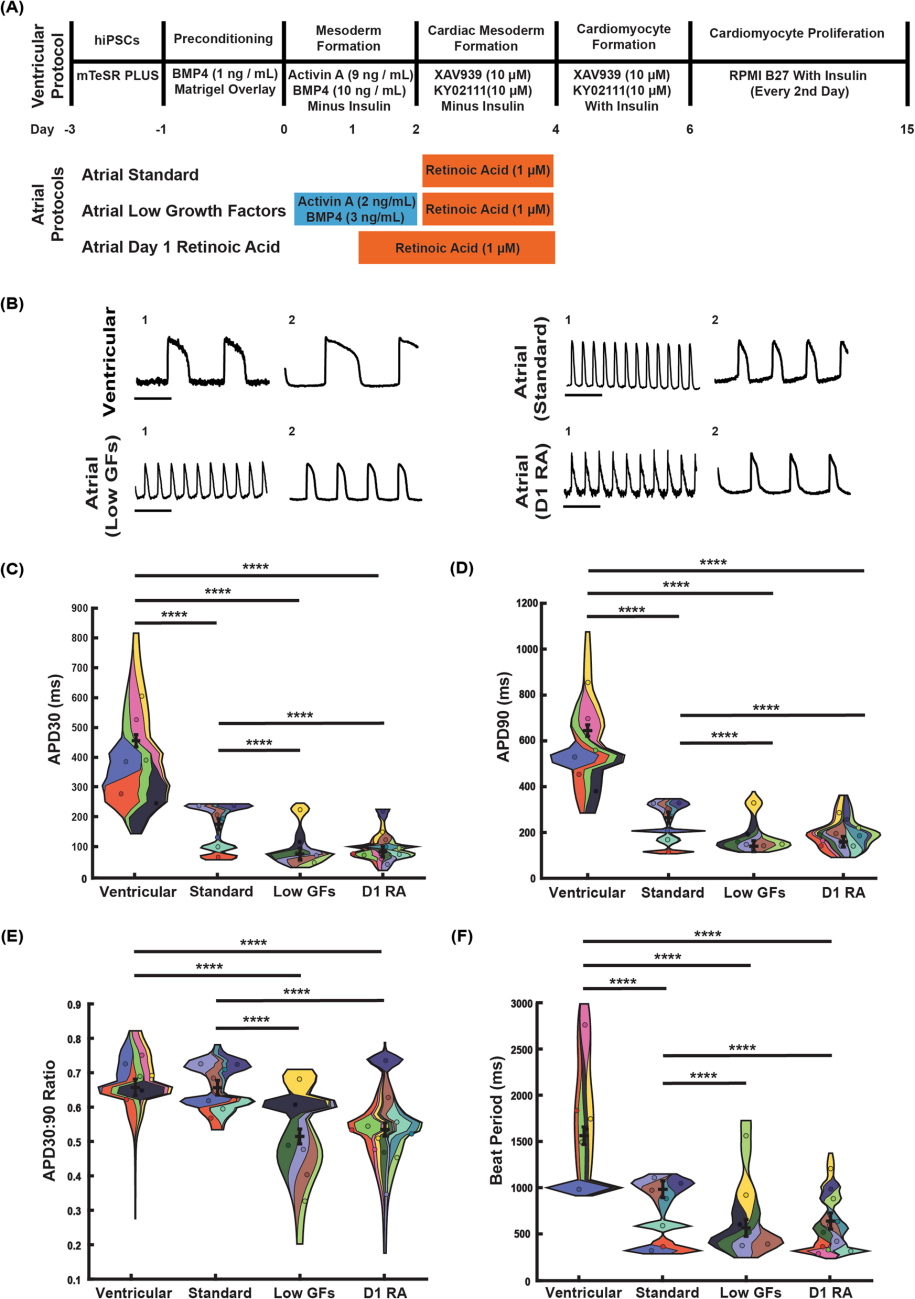
Derivation and action potential characterization of ventricular and atrial iPSC-derived cardiomyocytes. **A** Protocols for deriving ventricular and atrial cardiomyocytes from iPSCs, including three variations on atrial differentiation (standard, low growth factors (Low GF), and day 1 RA (D1RA)) applied to the base ventricular protocol. **B** Example action potential traces from two separate differentiations (1, 2) for each protocol; highlighting variability between differentiations. Time scale = 1 s. Summary of spontaneous action potential duration (APD) properties: **C** APD390, **D** APD90, **E** ratio of APD30 to APD90, **F** beat period. Each color represents a separate matched differentiation displayed as a violin plot, and individual violin plots are combined into superviolin plots (see methods for detailed explanation). Statistics performed with a mixed effects model. *N*/*n* = independent differentiations/technical repeats for ventricular (6/32), Atrial Standard (7/48), Atrial (Low GFs) (6/51), Atrial (D1RA) (11/85). ****p < 0.0001

Cardiomyocytes were produced using published protocols [31, 32]. In short, hiPSCs were TryPLE (12605010, ThermoFisher) dissociated and seeded at 105 k cells/cm^2^ in 10 μM ROCK inhibitor Y-27632 (72304, STEMCELL Technologies). Upon reaching 60–80% confluence, hiPSCs were preconditioned at day − 1 in RPMI B27 minus insulin (A1895601, ThermoFisher) with 2 ng/mL BMP4 (PHC9531, ThermoFisher), 1% GlutaMAX (35050079, ThermoFisher), 200 μM l-ascorbic acid (A15613.22, ThermoFisher), and 1:100 Matrigel for 16 h. Subsequently at day 0, hiPSCs were further induced to mesoderm in RPMI B27 minus insulin supplemented with 8 ng/mL Activin A (PHC9564, ThermoFisher) and 10 ng/mL BMP4 with 1% GlutaMAX. Day 2 cardiac mesoderm specification is induced with RPMI B27 minus insulin, 200 μM l-ascorbic acid, 10 μM KY02111 (4731, TOCRIS), and 10 μM XAV939 (X3004, Sigma) for 48 h before switching to the same formulation in RPMI B27 plus insulin for a further 48 h. Day 6 onward, differentiations were medium exchanged every 48 h for RPMI B27 plus insulin (1504001, ThermoFisher) supplemented with 200 μM l-ascorbic acid, until ready for dissociation day 12–15 (see Fig. 1).

For atrial conditions, the above protocol was adjusted as follows. Atrial (D1RA) differentiation included the addition of 1 μM retinoic acid (R2625, Sigma) at day 1 of differentiation (midway through mesoderm formation), followed by the addition of 1 μM retinoic acid at day 2 of differentiation (cardiac mesoderm formation). The Atrial Standard condition had the addition of 1 μM retinoic acid at day 2 of differentiation (cardiac mesoderm formation). Atrial (Low GFs) had reduced concentrations of Activin A and BMP4 to 2/3 ng /mL respectively during mesoderm formation, and 1 μM retinoic acid at day 2 of differentiation (see Fig. 1).

Cardiac myocyte dissociation was carried out with 0.2% collagenase type I (17018029, ThermoFisher), in PBS supplemented with 20% fetal bovine serum (FBS) (SH30084.04, Cytiva Life Sciences), for 45 min at 37 °C, followed by centrifugation at 300*g* for 3 min. Cardiomyocytes were resuspended in 0.25% Trypsin with EDTA (25200056, ThermoFisher) for 10 min at 37 °C, neutralized in FBS containing medium and filtered through a 70 µm cell strainer (352350, Corning), centrifuged at 300*g* for 3 min, and resuspended in B27 plus insulin supplemented with 10% FBS. After 48 h of seeding, medium was exchanged for B27 plus insulin, without FBS.

### Immunocytochemistry

Cells were fixed in 4% paraformaldehyde (PFA) (C004, ProSciTech) for 15 min at room temperature before washing × 3 in PBS, and permeabilizing/blocking in blocking buffer (PBS with 0.1% Triton-X100 (X100, Sigma) and 4% goat serum (G9023, Sigma)) for 1 h. Incubation with primary antibodies diluted in blocking buffer was carried out overnight at 4 °C. Primary stained samples were washed in PBS × 3 before addition of secondary antibodies in blocking buffer for 1 h at room temperature, exchanged to PBS containing DAPI (D9542, Sigma) for 10 min, washed in PBS × 3 and stored in PBS. Antibodies and concentrations used are provided in Additional file 1: Table S1.

Imaging was carried out using either an Opera Phenix (PerkinElmer) spinning disk confocal microscope (equipped with 2 × 16-bit sCMOS cameras), or LSM900 confocal microscope (Zeiss). Identification of objects, fluorescent intensity calculations, and percentage positivity was carried out using Harmony software (PerkinElmer).

### Cardiomyocyte and bead size flow cytometry

The proportion of Live hiPSC-derived cardiomyocytes were determined by staining the cells with Zombie Yellow™ Fixable Viability Kit (1:1000, 423103, BioLegend), Propidium iodide (1 µg/mL, P4170-10MG, Sigma-Aldrich), Calcein AM (1 µM, C3100MP, LifeTechnologies), and DAPI (10 µg/mL, D9542-10MG, Sigma-Aldrich) in PBS. The stained samples were analyzed using a CytoFLEX Flow Cytometer (Beckman Coulter).

Cardiomyocytes were fixed with 2% PFA for 10 min at room temperature. Cells were washed once with 500 μL of BD perm/wash buffer, incubated for 20 min, and then centrifuged (500×*g* for 5 min). Cardiomyocytes were incubated with Troponin T (1:500 dilution, MS295P1, ThermoFisher) for 1 h at room temperature in BD perm/wash buffer. Cardiomyocytes were then washed three times with PBS and then incubated with a secondary antibody for 1 h (1:500 dilution, A-21037, ThermoFisher). Cells were subsequently washed twice with PBS and then stained with Hoechst 33342 (Hoechst 33342, bisBenzimide H 33342 trihydrochloride, B2261-25MG, Sigma-Aldrich) prepared in perm/wash buffer.

Samples were analyzed using a CytoFLEX Flow Cytometer (Beckman Coulter). 20,000 events were collected for each experiment. Data were collected using CytExpert Software. Flow cytometry grade microbeads were obtained from Spherotech (PPS-6K). 1000–2000 microbeads were recorded for each bead size using identical parameters used for cardiomyocytes. All flow cytometry data were analyzed using FlowJo software [Becton Dickinson & Company (BD)]. Cell size measurements were obtained using the linear equation and formula obtained from plotting bead size against forward scatter area (FSC-A). In Additional file 3: Fig. S2 bead size (µm) vs FSC-A was plotted using Prism (Version 9.0.0, GraphPad Software, LCC).

### Action potential and calcium transient recordings

To record the action potentials of cardiomyocytes, cells were loaded with 1× FluoVolt dye as per manufacturer's instructions (F10488, ThermoFisher) and exchanged into phenol red free RPMI (11835030, ThermoFisher), supplemented to 1 mM calcium chloride. For calcium transient recordings, Cal 520 AM dye (21130, AAT Bioquest) was incubated at 2.5 μM for 30 min at 37 °C. Cardiomyocytes were placed in the Nikon Eclipse Ti2-E Inverted Microscope, fitted with a Nikon Plan Fluor 10× objective (NA, 0.3) and imaged using an Andor Zyla sCMOS (Oxford Instruments) high-speed camera. Data were collected at 5 ms temporal resolution from regions of 512 × 512 pixels. Before recording, cells were placed into the live chamber (37 °C and 5% CO_2_) and left to equilibrate for 30 min. Data were processed utilizing an in-house MATLAB script [33].

### Electrical field potential measurements (MEA)

Electrical recordings of field potentials from multilayered sheets of cardiomyocytes were performed using a Maestro-APEX multi-electrode array (MEA) system (Axion Biosystems). Cultures were seeded on CytoView (M768-tMEA-48B, M384-tMEA06W, Axion Biosystems) or E-stim 48-well MEA plates (Axion Biosystems). Spontaneous recordings were taken using AxIS v2.5.1.10 software (Axion Biosystems) before processing with the CiPA tool. For drug experiments, identical golden electrodes were selected across recordings. All experiments were performed at 37 °C and 5% CO_2_.

### Reverse transcriptase (RT) quantitative PCR (RT-qPCR)

Total mRNA was isolated using TRIzol (15596026, ThermoFisher). RNA samples were processed with miRNeasy kit (217084, Qiagen), and cDNA was generated using SuperScript™ IV reverse transcriptase including ezDNase (11766050, ThermoFisher), for digestion of genomic DNA, both following manufacturer’s instructions. RT-qPCRs were performed in triplicate using Lightcycler 480 SYBR Green master mix I (04707516001, Roche) in a CFX384 Optical Reaction Module on C1000 Touch Thermal Cycler (Bio-Rad). Expression data were analyzed using 2^−ΔΔCT^ method relative to expression level of GAPDH housekeeper gene. RT-qPCR analysis was performed on age matched differentiations produced simultaneously, across two different iPSC lines. Primers sequences are contained in Additional file 1: Table S2.

### Phase mapping and phase singularity detection

Optical recordings were first processed using the *Sliding Window Normalization* technique as described in [34], using a window size of 100 frames, to amplify optical signals. Further normalization methods outlined in [35] were used for spatial and temporal de-noising. Frames were passed through a Gaussian kernel filter before optical signals were filtered using a 4th-order Butterworth filter with a 1–30 Hz bandpass applied in forward and reverse mode. Signal edge tapering was achieved using an Hanning window, and signal smoothing was accomplished with sinusoidal wavelet recomposition.

Phase mapping and phase singularity detection was conducted following the transforms and calculations laid out by [35] and [36]. The Hilbert transform was utilized to obtain the instantaneous phase for the optical data. Phase singularities were detected using the *Double Ring Method* as described in [35] where a singularity is present when phase difference around a point of interest is greater than pi.

### Statistical analysis

For comparison of differentiation methods, data were fitted with a mixed effect model using the *glmfit* function in MATLAB, with differentiation method assigned as a fixed categorical variable and differentiations (*N*) and technical replicates (*n*) as random variables. Estimated marginal means were derived from the generalized linear mixed effect models using the *emmeans* package [37] with comparison between differentiations undertaken using a Wald test in input contrasts. Violin plots of data were created using Violin SuperPlots in MATLAB [38]. Error bars on violin plots represent estimated marginal means from the generalized linear mixed model and their standard errors. Datasets and code for statistical analysis and data visualization are available for download from Zenodo (https://zenodo.org/record/8145517). Values presented as estimated marginal means obtained by generalized linear mixed model and their standard errors.

## Results

### Tuning of retinoic acid and growth factor addition promotes atrial-like electrophysiology of iPSC-derived cardiomyocytes

To refine the monolayer method for generating atrial-like cardiomyocytes, three strategies were trialed (Fig. 1A). First was the addition of 1 μM retinoic acid during cardiac mesoderm formation to an established protocol for generating ventricular cardiomyocytes, the most common method for production of atrial-like cardiomyocytes reported in the literature [18, 23–25], herein referred to as Atrial Standard. Second, we modified Atrial Standard by reducing growth factor addition during mesoderm formation, mimicking the protocol developed by [27] but without cell sorting as described in [16], herein referred to as Atrial (Low GFs). Third, we sought to enhance atrial-like cardiomyocyte production through the addition of 1 μM retinoic acid on day 1 (midway during mesoderm formation), as well as on day 2, which we term Atrial (D1RA). For comparison, we also generated ventricular myocytes using the protocol published by [31, 32].

Typical examples of action potentials recorded for each atrial differentiation method (~ 30 days post-differentiation) are shown in Fig. 1B. All three protocols produced cardiomyocyte action potentials (AP) with less pronounced plateau phases, shorter spontaneous beat periods, and shorter AP duration measured at 30% repolarization (APD30) and 90% repolarization (APD90), and smaller APD30:APD90 ratios, compared to ventricular differentiations (Fig. 1C–F and Table 1). However, differences were observed between protocols. Cardiomyocytes derived using Atrial (Low GFs) or Atrial (D1RA) had shorter beat periods and shorter APD30 and APD90 values compared to the Atrial Standard method. Additionally, APD30:90 ratio, a key indication of atrial-like AP morphology [39, 40], was significantly lower in Atrial (Low GFs) cardiomyocytes (0.52 ± 0.02) and Atrial (D1RA) cardiomyocytes (0.54 ± 0.02) than Atrial Standard cardiomyocytes (0.66 ± 0.02). Indeed, cardiomyocytes produced using the Atrial Standard protocol had APD30:90 ratios that were like those of ventricular differentiations (Table 1). Thus, Atrial (D1RA) and Atrial (Low GFs) produced myocytes that had more atrial-like action potential properties than Atrial Standard.

**Table 1:** Summary of action potential parameters for different differentiation protocols

Action potential parameters (*N*/*n*)	Average beat period (ms)	Average APD30 (ms)	Average APD90 (ms)	Average APD30:90 ratio
Ventricular (6/32)	1561 ± 95	455 ± 20	645 ± 25	0.66 ± 0.02
Atrial Standard (7/48)	983 ± 91	176 ± 19	265 ± 24	0.66 ± 0.02
Atrial (Low GFs) (6/51)	564 ± 91	76 ± 19	140 ± 24	0.52 ± 0.02
Atrial (D1RA) (11/85)	639 ± 87	84 ± 18	160 ± 23	0.54 ± 0.02

*N* = Experimental replicates (separate differentiations), *n* = technical replicates. Values presented as estimated marginal means obtained by generalized linear mixed model and their standard errors

Importantly, for the Atrial (Low GFs) and Atrial (D1RA) protocols, cells retained their atrial-like properties after 100 days in culture, indicating that atrial-like properties of the cells after 30 days differentiation are not simply a reflection of immaturity. For example, APD90 values for Atrial (Low GFs) and Atrial (D1RA) after 100 days differentiation was 126 ms and 129 ms respectively, and APD30:90 ratios were 0.42 and 0.41, respectively (see Additional file 2: Fig. S1). Conversely, for the Atrial Standard protocol, after 100 days differentiation, cells displayed a more ventricular-like phenotype with APD90 value of 344 ms and APD30:APD90 ratio of 0.71.

### Retinoic acid addition alters tissue-level electrical properties of iPSC-derived cardiomyocytes

**Fig. 2:**
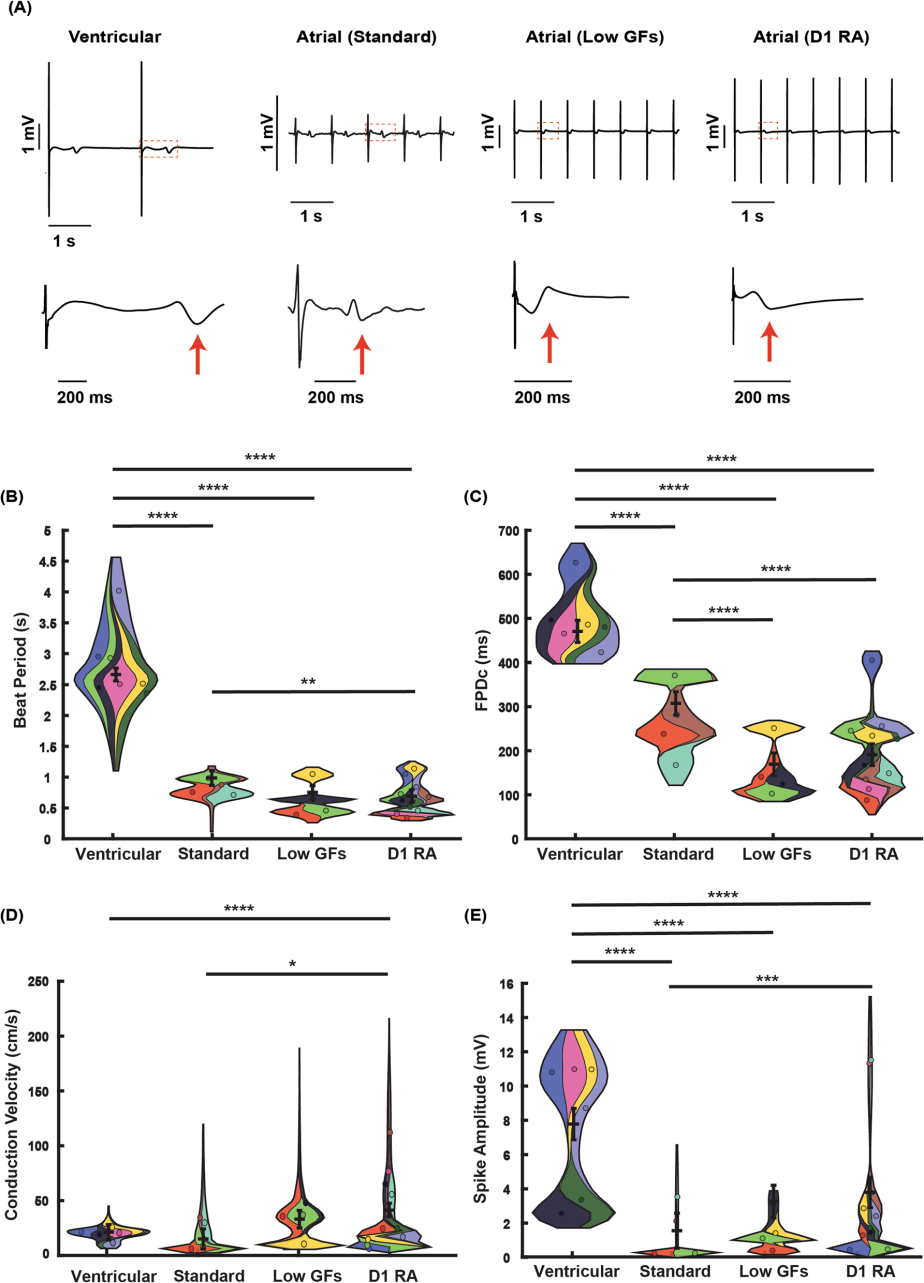
Multi-electrode array (MEA) characterization of spontaneous ventricular and atrial iPSC-derived cardiomyocyte field potentials. **A** The electrical properties of syncytium produced by each differentiation protocol were compared utilizing a MEA, with representative field potential traces from MEA recordings displayed. Short traces (4 s) and single FPD traces are shown. The red arrow indicates position of FPD measurement. Summary of MEA recordings: **B** beat period, **C** field potential duration, corrected for beat period using Fredericia’s formula, **D** spike amplitude, and **E** conduction velocity. Statistics performed with a mixed effects model. *N*/*n* = independent differentiations/technical repeats for ventricular (6/69), Atrial Standard (4/24), Atrial (Low GFs) (4/38), Atrial (D1RA) (10/107). *p < 0.05, **p < 0.01, ***p < 0.001, ****p < 0.0001

We next investigated the electrical properties (field potentials) of cultured monolayers of cardiac cells produced from each differentiation protocol. Typical extracellular electrograms recorded from each differentiation method are shown in Fig. 2A. All atrial differentiation protocols produced monolayers with shorter beat periods (Fig. 2B) and rate corrected field potential durations (FPDc) (Fig. 2C), compared to ventricular differentiations. Furthermore, consistent with optical action potential measurements (see Fig. 1), shorter FPDc values were seen in Atrial (Low GFs) (168 ± 25.6 ms) and Atrial (D1RA) (190 ± 24.8 ms) protocols, compared to Atrial Standard (307 ± 26.5 ms) (Fig. 2B).

Conduction velocity measurements for ventricular myocyte monolayers were consistent across independent differentiations (20.2 ± 7 cm s^−1^, *N* = 6, Fig. 2D). However, conduction velocities in atrial monolayers varied between differentiations. For Atrial (Low GFs) and Atrial (D1RA) monolayers, conduction velocities ranged from < 10 to > 50 cm s^−1^ (Fig. 2D). Conduction velocities in Atrial Standard monolayers tended to be slower than seen for other methods but were only significantly slower than Atrial (D1RA) monolayers, with no statistical difference from ventricular or Atrial (Low GFs) differentiations (Fig. 2D).

Electrogram spike amplitudes—reflecting the propagating action potential upstroke—were significantly smaller in cardiomyocyte monolayers produced by atrial differentiation methods compared to ventricular differentiations. There was also significant variation between differentiations for atrial protocols. The three atrial protocols produced monolayers with spike amplitudes clustered in the < 1 mV and 1–5 mV range, but only the Atrial (D1RA) method produced spike amplitudes exceeding 8 mV (Fig. 2E). There was no correlation between spike amplitudes and beat periods or FPDc values in atrial monolayers. Differentiations that produced the highest spike amplitudes for Atrial (D1RA) had beat periods < 0.6 s and FPDc values < 150 ms, indicating the potential to create superior atrial-like phenotypes.

Through a combination of enhanced conduction velocity and spike amplitudes, Atrial (D1RA) produced the most functionally appropriate phenotype out of the methods tested (Table 2).

**Table 2:** Summary of multi-electrode array (MEA) recordings across protocols

Multi-electrode array parameters (*N*/*n*)	Median beat period (s)	Median FPDc (ms)	Median spike amplitude (mV)	Median conduction velocity (cm s^−1^)
Ventricular (6/69)	2.66 ± 0.10	471 ± 25.2	7.8 ± 0.92	20.2 ± 7.0
Atrial Standard (4/24)	0.99 ± 0.12	307 ± 26.5	1.5 ± 1.02	13.9 ± 9.2
Atrial (Low GFs) (4/38)	0.75 ± 0.11	168 ± 25.6	3.2 ± 0.96	32.2 ± 8.0
Atrial (D1RA) (10/107)	0.69 ± 0.09	190 ± 24.8	3.8 ± 0.89	40.4 ± 6.3

*N* = Experimental replicates, *n* = technical replicates. Values presented as estimated marginal means obtained by generalized linear mixed model and their standard errors

### Cardiomyocytes display atrial-like pharmacology

**Fig. 3:**
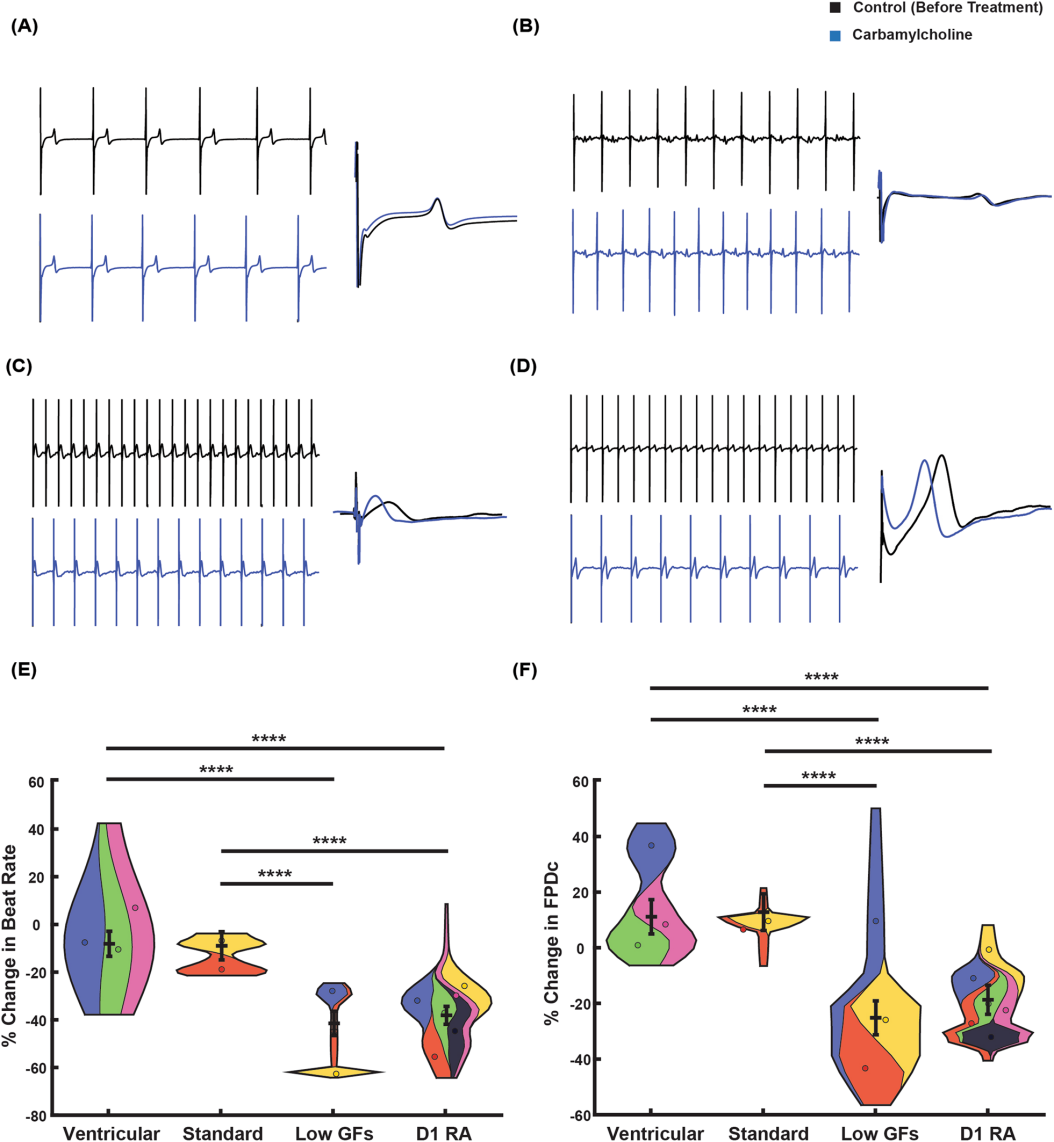
Pharmacological activation of *I*_kach_ current with muscarinic receptor agonist carbamylcholine. Representative 10 s traces recorded from a multi-electrode array, before (black), and after carbamylcholine (10 μM) exposure (blue), demonstrate the change, or lack of change, in beat period. Single FPD traces are overlaid for ventricular (**A**), Atrial Standard (**B**), Atrial (Low GFs) (**C**), and Atrial (D1RA) (**D**), demonstrating expected FPD shortening induced by carbamylcholine in Atrial (Low GFs) and Atrial (D1RA). Summary of changes in beat period (**E**) and corrected field potential duration (**F**) after carbamylcholine exposure, values adjusted to vehicle controls. Statistics performed with a mixed effects model. *N*/*n* = independent differentiations/technical repeats for ventricular (3/11), Atrial Standard (2/9), Atrial (Low GFs) (3/12), Atrial (D1RA) (6/28). ****p < 0.0001

We next assessed how differentiated cardiomyocytes responded to carbamylcholine, a muscarinic receptor agonist that activates atrial/nodal specific G protein-coupled potassium channels (*I*_kach_). Example traces from identical electrodes on a multi-electrode array (MEA), before and after addition of carbamylcholine (10 µM), are shown in Fig. 3A–D. For the ventricular and Atrial Standard differentiations, carbamylcholine had no significant effect on the vehicle adjusted beat rate or field potential duration. Conversely for Atrial (Low GFs) and Atrial (D1RA) protocols, both beat period and FPDc were reduced, indicating the presence of functional *I*_kach_ currents in these preparations (Fig. 3E–F).

### Atrial (low GFs) fails to routinely form cardiac monolayers, atrial (D1RA), and atrial standard are robust in cardiac monolayer formation

Atrial standard and atrial (D1RA) were robust protocols achieving successful beating monolayers of atrial cardiomyocytes in 9/10 and 16/18 independent differentiations, respectively. The atrial (Low GFs) protocol, however, often failed to form a confluent mesoderm with only 8/17 successful independent differentiations as assessed by the presence of beating myocytes by day 13–15 of differentiation.

### Atrial-like cardiomyocytes lack MLC2v protein expression and have an atrial-like gene expression profile

Immunostaining of day 30 atrial differentiated cultures showed high levels of MLC2a expression and greatly reduced MLC2v levels, compared to myocytes derived using the ventricular protocol (Additional file 3: Fig. S2A), with further independent experiments identifying low levels of MLC2v positive ventricular cells present in the syncytia (Additional file 3: Fig. S2B). Atrial (D1RA) protocol produced cells with an atrial-like gene expression profile, with reduced MYL2 and MYH7 but increased NR2F2, KCNA5 and KCNJ3 expression, analyzed by qRT-PCR (Additional file 3: Fig. S2C).

### Atrial (D1RA) protocol is robust and generates smaller cTnT+ cells than the ventricular protocol

Atrial (D1RA) protocol proved to be the most robust at generating atrial-like cardiomyocytes and was carried forward for more in-depth examination. Freshly dissociated cells from Atrial (D1RA) protocols were significantly smaller than cells from ventricular dissociations (Additional file 4: Fig. S3A i–iii). The proportion of cTnT+ cells among fixed cells pooled from three separate matched differentiations were 88.2% and 84.8% for ventricular and Atrial (D1RA) protocols, respectively (Additional file 4: Fig. S3B, C). The fixed cTnT+ cells from the Atrial (D1RA) differentiations were also significantly smaller than the fixed cells from the ventricular differentiations (7.9 µm vs. 13.4 µm, respectively), similar to what has been reported elsewhere [41, 42], with relative profiles similar to freshly dissociated cells (Additional file 4: Fig. S3A, D).

### Atrial (D1RA) protocol could be applied to 6 iPSC lines without modification

**Fig. 4:**
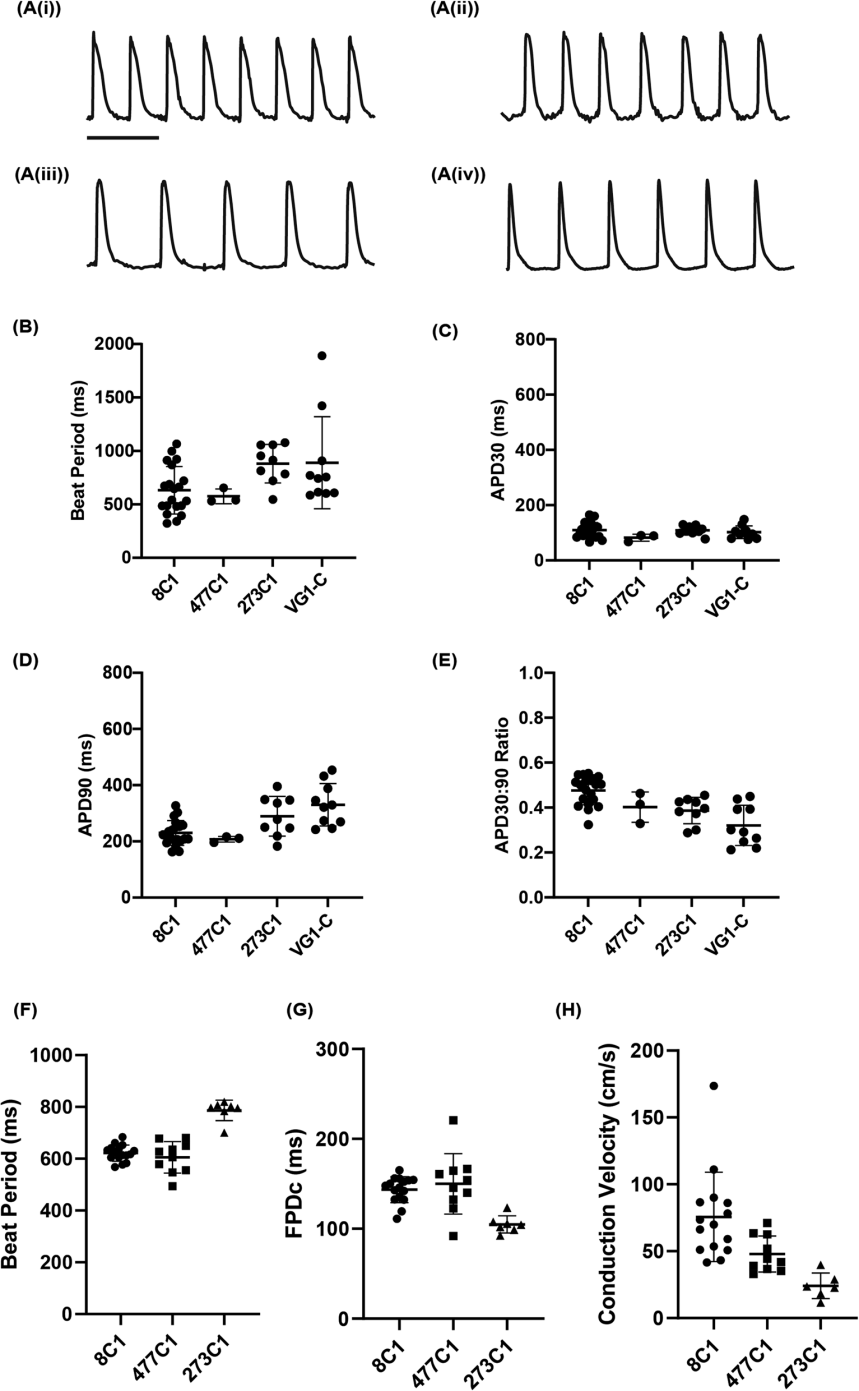
Atrial (D1RA) protocol could be used on four additional iPSC lines without re-optimization. Four additional iPSC lines were differentiated without optimization and examined for their electrical properties. Example action potential traces are shown in (**A**(i–iv)) for lines 8C1, 473C1, 273C1, and VG1-C, respectively. Scale bars = 1 s. Summary of quantified action potential recordings, **B** beat period, **C** APD30, **D**, APD90, **E** APD30:90 ratio. Each cell line was monitored for electrical properties on a multi-electrode array with, **F** beat period, **G** corrected field potential duration, and **H** conduction velocity quantified. *N*/*n* = independent differentiations/technical repeats for 8C1 (4/19), 473C1 (1/3), 273C1 (1/9), VG1-C (3/10). Error bars are ± 1 SD

We next examined the robustness of the Atrial (D1RA) protocol by applying it to an additional four iPSC lines, VG1-C, 8C1, 477C1, and 273C1. These four iPSC lines generated contractile syncytia that demonstrated atrial-like action potentials with APD90 values of 330 ± 71.3, 230 ± 43, 208 ± 8.2, and 289.6 ± 66.6 ms (mean ± SD), respectively (Fig. 4C). The slightly longer spontaneous APD90s compared to initially analyzed lines 15C1 and 100C1 (Figs. 1 and 2) could be explained by differences in beat period (Fig. 4A, D). All lines produced APD30:90 ratios < 0.5 (VG1-C 0.32 ± 0.09, 477C1 0.40 ± 0.06, 273C1 0.39 ± 0.06, 8C1 0.48 ± 0.06), thus demonstrating triangular AP morphology (Fig. 4E i–iv). Lines 8C1, 477C1, and 273C1 were additionally assessed on a MEA. FPDc values were 144 ± 14 (8C1), 150 ± 32 (477C1), and 105 ± 9 ms (273C1), respectively (Fig. 4G). Conduction velocities were rapid for lines 8C1 (75.6 ± 32 cm s^−1^) and 477C1 (47.9 ± 13 cm s^−1^) but slightly lower for line 273C1 (24.1 ± 8.8 cm s^−1^), with no correlation to beat period or FPDc (Fig. 4H). Thus, the Atrial (D1RA) protocol produced atrial-like phenotypes across 6 different iPSC lines.

### Rotor formation could be induced with burst pacing protocols

**Fig. 5.**
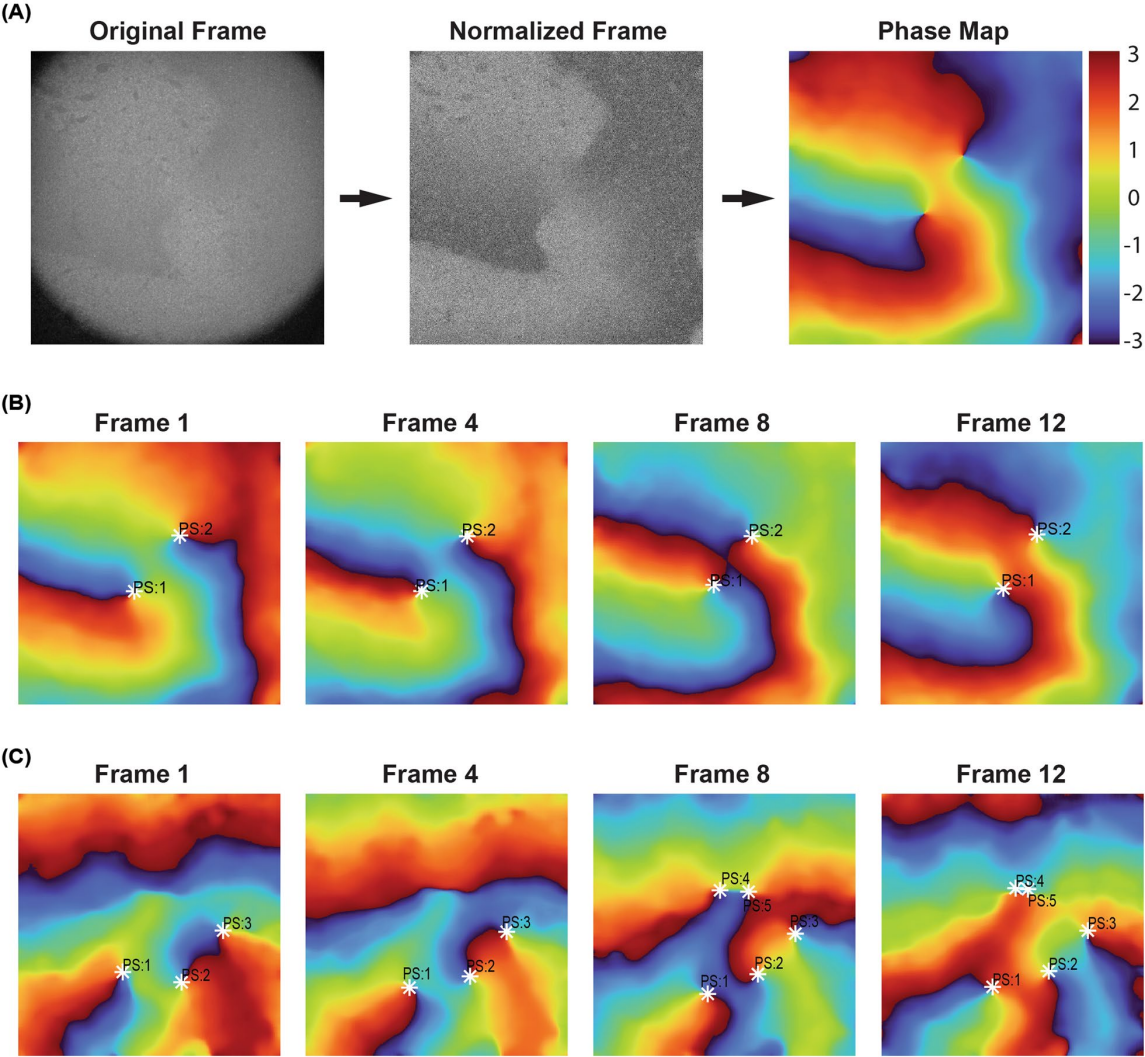
10 Hz burst pacing induced rotor formation with varying levels of complexity. 6-well atrial monolayers were paced at 10 Hz for 30 s to induce reentrant arrhythmic activity. **A** Optical maps of calcium transients were converted into phase maps utilizing a frame normalization process in MATLAB™. Individual frames of converted phase maps are sequentially displayed, with phase singularities identified, for a dual phase singularity recording and a complex multi-phase singularity recording, respectively, in (**B**) and (**C**)

We next examined whether reentrant arrhythmias could be induced in monolayers of atrial cardiomyocytes using tachypacing to demonstrate the applicability of our atrial cells to modelling atrial fibrillation in vitro. Tachypacing elicited stable rotors with varying degrees of complexity in different monolayers (Additional file 5: Video S1 and Additional file 6: Video S2) that were visualized using phase mapping (Fig. 5A). An example of a simple rotor pattern is shown in Fig. 5B/Additional file 7: Video S3. In this case, two adjacent and stable phase singularities (PS) exist that eventually collide before renewal of rotors occurs in the same location. In this recording stable rotors lasted 22,000 ± 0 ms, and wavefronts existed for 61.5 ± 90 ms. In comparison, Fig. 5C/Additional file 8: Video S4 illustrates a much more complex, multi-PS arrhythmia, characterized by > 500 transient PS, with a mean singularity lifetime of 1474 ± 1639 ms and mean wavefront lifetime of 71.2 ms ± 90 ms, that migrated across the sample. The ability to form reentrant rotors demonstrates the utility of iPSC atrial cardiomyocytes in future anti-arrhythmic drug screens.

## Discussion

We have established an economical, robust, and scalable methodology to create iPSC atrial-like cardiomyocytes. To keep the methodology simple and scalable, all methods compared were monolayer based, avoiding the laborious formation of embryoid bodies used in many existing protocols [16, 24, 25, 27, 28, 43]. The use of commercial kits was not considered; whereas they have been successfully used by others [26, 44, 45], as components are proprietary, and they add significant cost per differentiation, additionally these methods require extensive lactate purification, reducing yield. The use of genetic reporter lines and/or FACs to enrich atrial-like populations has been successful in generating relatively pure atrial cardiomyocytes [27, 46], but this approach limits scalability beyond the genetically edited cell lines and adds additional costs for FACs-related methods and handling.

For optimization, we replicated retinoic acid addition to cardiac mesoderm formation protocols, Atrial Standard, as well as a reduced growth factor protocol, Atrial (Low GFs), and explored the possibility of maximizing mesoderm populations programmed for atrial myocyte formation by adding retinoic acid during the mesoderm formation stage (Atrial D1RA), a method not previously published.

We performed a comparison of each methodology based on functional assays: AP assessment, MEA field potential recordings, and response to carbamylcholine. Across AP and FP data all methods produced cells that had significantly faster beating rates, shorter APs, and FPDc values compared to the ventricular differentiation protocol, in agreement with existing studies [24–27]. Atrial (Low GFs) and Atrial (D1RA) produced shorter APD90 values in matched differentiation sets than Atrial Standard, which were broadly in line with the 100–200 ms APD90 values reported in embryoid body-based, low growth factor methods [27].

Human adult atrial cardiomyocytes isolated from patients, paced at 1 Hz, are heterogeneous in their APDs with APD90s ranging from 190 to 440 ms, meaning APD90 values alone were insufficient to determine atrial phenotype [47]. APD30:90 ratios for cardiomyocytes produced using Atrial (Low GFs) and Atrial (D1RA) protocols were significantly lower than for ventricular myocytes, while the Atrial Standard protocol did not produce low APD30:90 ratios, suggesting insufficient *I*_Kur_ and *I*_kach_ currents, which was confirmed by lack of response to carbamylcholine. An in-depth single cell sequencing study of different mesoderm progenitors and the resulting cardiac populations generated showed that high BMP4 + RA can lead to the formation of right ventricular cardiomyocytes, which could potentially explain why Atrial Standard showed reduced APDs versus the ventricular protocol but maintained a broad APD30:90 ratio [28]. Atrial (Low GFs) and Atrial (D1RA) have comparable APD30:90 values (0.52 ± 0.02 and 0.54 ± 0.02) that align with atrial-like cardiomyocytes previously reported [16, 25], but have broader AP morphologies than those reported in human adult atrial cardiomyocytes [47].

Field potential measurements from MEA experiments aligned with data from action potential recordings. FPDc values in Atrial (Low GFs) and Atrial (D1RA) syncytium were significantly shorter than those produced by the Atrial Standard protocol. Ventricular cardiomyocytes produced the greatest spike amplitudes; however, out of the atrial protocols examined, only Atrial (D1RA) produced spike amplitudes significantly higher than Atrial Standard, which align with those reported by [45]. These results suggest that the Atrial (D1RA) protocol can produce cardiomyocytes with enhanced upstroke properties during depolarization.

Lines 100C1, 15C1, 8C1, and 477C1 produced remarkably high monolayer conduction velocities for both Atrial (Low GFs) and Atrial (D1RA) protocols (Figs. 2D and 4H). Conduction velocity values regularly exceeded 40 cm s^−1^ and sometimes surpassed 90 cm s^−1^, far beyond ~ 2.5–5.6 cm s^−1^ reported by [16, 48, 49] in iPSC atrial cardiomyocytes, and those reported in immortalized human atrial myocyte lines of ~ 20 cm s^−1^ [49]. The base ventricular protocol also produced enhanced conduction velocities of 20.2 ± 7.0 cm s^−1^ versus the 3.5–18 cm s^−1^ reported in previous studies of iPSC-ventricular cardiomyocytes [48, 50–52]. CV values reported using the atrial (D1RA) and atrial (Low GFs) methods could reach human adult-like velocities, which can range from 40 cm s^−1^ in slower regions (e.g., floor of the right atrium), ~ 74 cm s^−1^ within bulk atrial tissue, and 110–177 cm s^−1^ within the conduction bundles [53]. These results highlight the importance of mesoderm stage interventions, be it lower growth factor concentrations or retinoic acid addition, in producing enhanced atrial-like phenotypes. The Atrial Standard protocol demonstrated a lesser atrial-like phenotype when examining the combined action potential and MEA data and was ruled out as an appropriate protocol for future applications.

Differentiations of the ventricular, Atrial Standard, and Atrial (D1RA) were robust and routinely produced contractile sheets by day 15 of differentiation, 9/10 and 16/18 for Atrial Standard and Atrial (D1RA), respectively. However, only 47% (8/17) of Atrial (Low GFs) differentiations were successful. The lack of cardiomyocyte formation from Atrial (Low GFs) occurred in experiments with successful Atrial Standard and Atrial (D1RA) differentiations from the same starting population of iPSCs, across both cell lines tested. This suggests that the outcome was protocol specific and not due to poor quality of the iPSCs, growth factor batch effects, or retinoic acid degradation. Atrial (Low GFs) often failed to form good monolayer coverage of mesoderm before the onset of cardiac mesoderm differentiation, which had a poor prognosis for day 15 success rates.

These findings align with published results, where differentiation of additional iPSC/hESC lines required extensive re-optimization of BMP4 and Activin A concentrations to generate an ALDH+ mesoderm that underwent FACS enrichment before continued differentiation into atrial cardiomyocytes [27, 28]. Growth factor concentrations of 2 ng/mL Activin A and 5 ng/mL BMP4 were successful in one hESC line, but re-optimization to 1 ng/mL Activin A and 4 ng/mL BMP4 (+ an additional Nodal/Activin A/TGFβ1 inhibitor SB-431542) was required in another [27]. Yang et al. demonstrated concentrations as low as 0.5 ng/mL Activin A and 3 ng/mL BMP4 were required to generate sufficient ALDH+ mesoderm, but even small increases to 1.5 ng/mL Activin A or 2.5 ng/mL Activin A diminished the generation of ALDH+ mesoderm progenitors [28]. The requirement to re-optimize conditions per-line was explained by differences in endogenous Nodal/Activin A signaling. Similar re-optimization and FACs could have been carried out within this study, with the potential to improve the robustness of the Atrial (Low GFs) protocol; however, this approach would severely limit the scalability beyond a small number of iPSC lines in future studies. In contrast, the Atrial (D1RA) protocol was readily applicable to 6 independent iPSC lines making it the most appropriate method to take forward as a scalable drug screening platform.

## Conclusions

We have developed a simple method to differentiate atrial cardiomyocytes that show faster beat rates, shortened and more triangular APs, reduced rate corrected FPDs, respond to carbamylcholine induced *I*_kach_ activation, atrial-like expression profiles, reduced cellular size, and near adult-like conduction velocities. This method demonstrated functional improvements compared to standard RA addition and improved robustness of differentiation compared to a low growth factor protocol. Additionally, this method was readily applicable to 6 iPSC lines without additional re-optimization, highlighting its potential for broad applicability. We could also elicit arrhythmic activity in response to burst pacing, establishing utility for future drug screening platforms and investigations into atrial cardiomyopathy.

## Supplementary Information

**Additional file 1: Table S1.** Antibodies for atrial versus ventricular assessment. **Table S2.** qPCR primers for assessment of cell identity, ventricular, and atrial gene expression profiles.

**Additional file 2: Fig. S1.** Action potential recordings from differentiation day 100 matured atrial cardiomyocytes. (**A**(i–iii)) MEA recordings from MEA Cytoview plates, amenable to imaging, subsequently assessed for voltage action potentials at day 100. Data displays a single field potential MEA trace followed by the corresponding action potential trace and data for, (**A**(i)) Atrial (D1RA), (**A**(ii)) Atrial Standard and (**A**(iii)) Atrial (Low GFs).

**Additional file 3: Fig. S2.** Molecular characterization of atrial and ventricular differentiation methods. **A** To determine the effectiveness of the atrial differentiation method to produce atrial but not ventricular cardiomyocytes, cultures underwent immunostaining for ventricular marker (MLC2v) and atrial marker (MLC2a). Scale bars = 100 μm. **B** Immunostaining for MLC2v and α-actinin presence in two further independent experiments for Atrial (D1RA). **C** qPCR analysis of Atrial (D1RA) compared to matched ventricular controls. Unpaired t-test statistical testing performed for qPCR samples.

**Additional file 4: Fig. S3.** Flow cytometry comparison of ventricular and atrial (D1RA) differentiation protocols. **A** Quantification of cell size from live populations, (i) comparison of forward and side scatter parameters of Atrial (D1RA) and ventricular protocol versus calibration beads of known size. (ii) Relationship between calibration bead size and forward scatter area parameter. (iii) Counted cells versus forward scatter. **B** and **C** Flow cytometry analysis for cTnT positive populations in ventricular and atrial (D1RA) protocols respectively, from three pooled independent experiments. **D** Quantification of cTnT+ populations for cell size from (**B**, **C**), against calibration beads of known size.

**Additional file 5: Video S1. **Normalised optical fluorescent recording of two stable stationary rotors induced by tachypacing.

**Additional file 6: Video S2.** Normalised optical fluorescent recording of a complex multi-rotor arrhythmia induced by tachypacing.

**Additional file 7: Video S3.** Phase map video of two stable stationary rotors induced by tachypacing.

**Additional file 8: Video S4.** Phase map video of a complex multi-rotor arrhythmia induced by tachypacing.

## Data Availability

The datasets generated and/or analyzed during the current study are available in the Zenodo depository (doi tbc).

## Abbreviations

AF: Atrial fibrillation
ALDH: Aldehyde dehydrogenase
AP: Action potential
APD: Action potential duration
BMP4: Bone morphogenic protein 4
CTNT: Cardiac troponin
CV: Conduction velocity
FACS: Fluorescence-activated cell sorting
FPD: Field potential duration
FPDc: Field potential duration corrected
HESC: Human embryonic stem cell
HiPSC: Human-induced pluripotent stem cell
IPSC: Induced pluripotent stem cell
KCNA5: Potassium voltage-gated channel subfamily A member 5
KCNJ3: Potassium inwardly rectifying channel subfamily J member 3
MEA: Multi-electrode array
MLC2a: Myosin light chain 2 atrial
MLC2v: Myosin light chain 2 ventricular
MYH7: Myosin heavy chain 7
MYL2: Myosin light chain 2
NR2F2: Nuclear receptor subfamily 2 group F member 2
QRT-PCR: Quantitative reverse transcriptase polymerase chain reaction
RA: Retinoic acid
PS: Phase singularity
TGFβ1: Transforming growth factor beta 1
