# Correction: Development of a robust induced pluripotent stem cell atrial cardiomyocyte differentiation protocol to model atrial arrhythmia

**DOI:** 10.1186/s13287-026-04942-5

**Published:** 2026-02-16

**Authors:** Jordan Thorpe, Matthew D. Perry, Osvaldo Contreras, Emily Hurley, George Parker, Richard P. Harvey, Adam P. Hill, Jamie I. Vandenberg

**Affiliations:** 1 Victor Chang Cardiac Research Institute, Sydney, NSW Australia; 2 Department of Pharmacology, School of Biomedical Sciences, University of New South Wales, Sydney, Australia; 3 School of Clinical Medicine, Faculty of Medicine and Health, University of New South Wales, Sydney, Australia; 4 School of Biotechnology and Biomolecular Science, University of New South Wales, Sydney, NSW Australia

**Correction: Stem Cell Research & Therapy (2023) 14:183**

10.1186/s13287-023-03405-5

The original article presents an error in Figure 1A—for the Preconditioning step, the text ‘1ng/mL’ should instead state ‘2ng/mL’.

