# Supplementary material for: Development of a robust induced pluripotent stem cell atrial cardiomyocyte differentiation protocol to model atrial arrhythmia
Source: Stem Cell Res Ther. 2023 Jul 27;14:183. doi: 10.1186/s13287-023-03405-5 (PMC10373292; doi:10.1186/s13287-023-03405-5)
Supplement: Supplementary file 1 — Additional file 1: Table S1. Antibodies for atrial versus ventricular assessment. Table S2. qPCR primers for assessment of cell identity, ventricular, and atrial gene expression profiles. [file 13287_2023_3405_MOESM1_ESM.docx]

**Supplementary table 1. Antibodies for atrial versus ventricular assessment.**

| **Target Name** | **Supplier** | **Product Code** | **Dilution** |
| --- | --- | --- | --- |
| Α-actinin | Sigma | A7811 | 1:800 |
| MLC2v | Proteintech | 10906-1-AP | 1:200 |
| MLC2a | Abcam | ab68086 | 1:1000 |

**Supplementary table 2. qPCR primers for assessment of cell identity, ventricular, and atrial gene expression profiles**

| **Gene Name** | **Forward Sequence 5’ – 3’** | **Reverse Sequence 5’ – 3’** |
| --- | --- | --- |
| GAPDH | CAG CCT CAA GAT CAT CAG CAA TG | CCA TCC ACA GTC TTC TGG GTG |
| MYL2 | GGC GAG TGA ACG TGA AAA AT | CAG CAT TTC CCG AAC GTA AT |
| MYH7 | AAA GAG GCG CTA GAG AAG TCC G | CAG CAT CTG CCA GGT TGT CTT G |
| NR2F2 | AGC AAG TGG AGA AGC TCA AGG C | TGG GCT ACA TCA GAG AGA CCA C |
| KCNA5 | CTG CTC ATC TTC TTC CTC TTC ATC G | TCA GGG ATG CTA GAG AAA TGG GTT C |
| KCNJ3 | GCA CGC GGT GAT CTC CAT GA | ACC CTC AGG TGT CTG CCG A |
