# Supplementary figures and images for: Development of a robust induced pluripotent stem cell atrial cardiomyocyte differentiation protocol to model atrial arrhythmia
Source: Stem Cell Res Ther. 2023 Jul 27;14:183. doi: 10.1186/s13287-023-03405-5 (PMC10373292; doi:10.1186/s13287-023-03405-5)

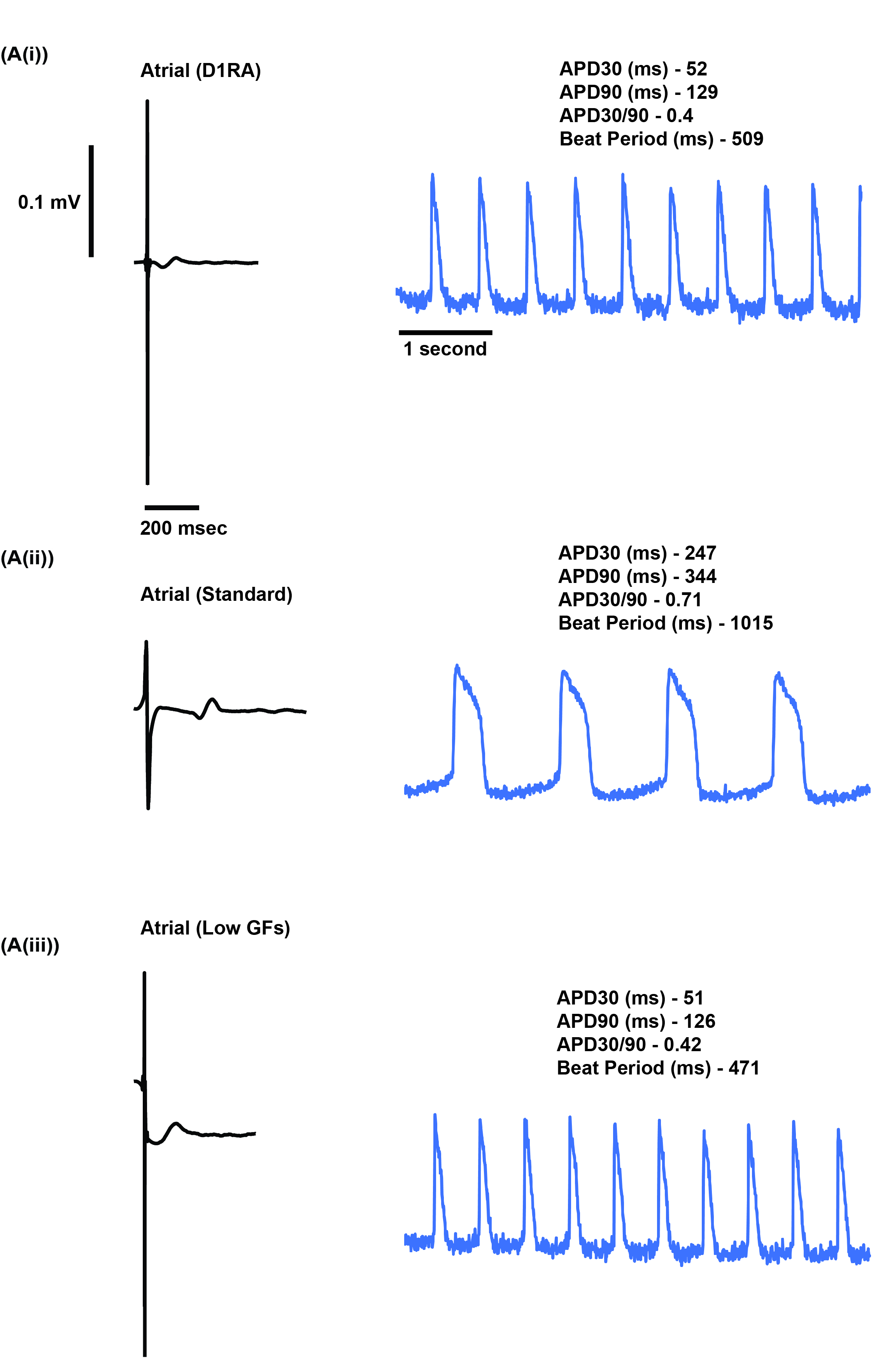

Supplement: Supplementary file 2 — Additional file 2: Fig. S1. Action potential recordings from differentiation day 100 matured atrial cardiomyocytes. (A(i–iii)) MEA recordings from MEA Cytoview plates, amenable to imaging, subsequently assessed for voltage action potentials at day 100. Data displays a single field potential MEA trace followed by the corresponding action potential trace and data for, (A(i)) Atrial (D1RA), (A(ii)) Atrial Standard and (A(iii)) Atrial (Low GFs). [file 13287_2023_3405_MOESM2_ESM.jpg]

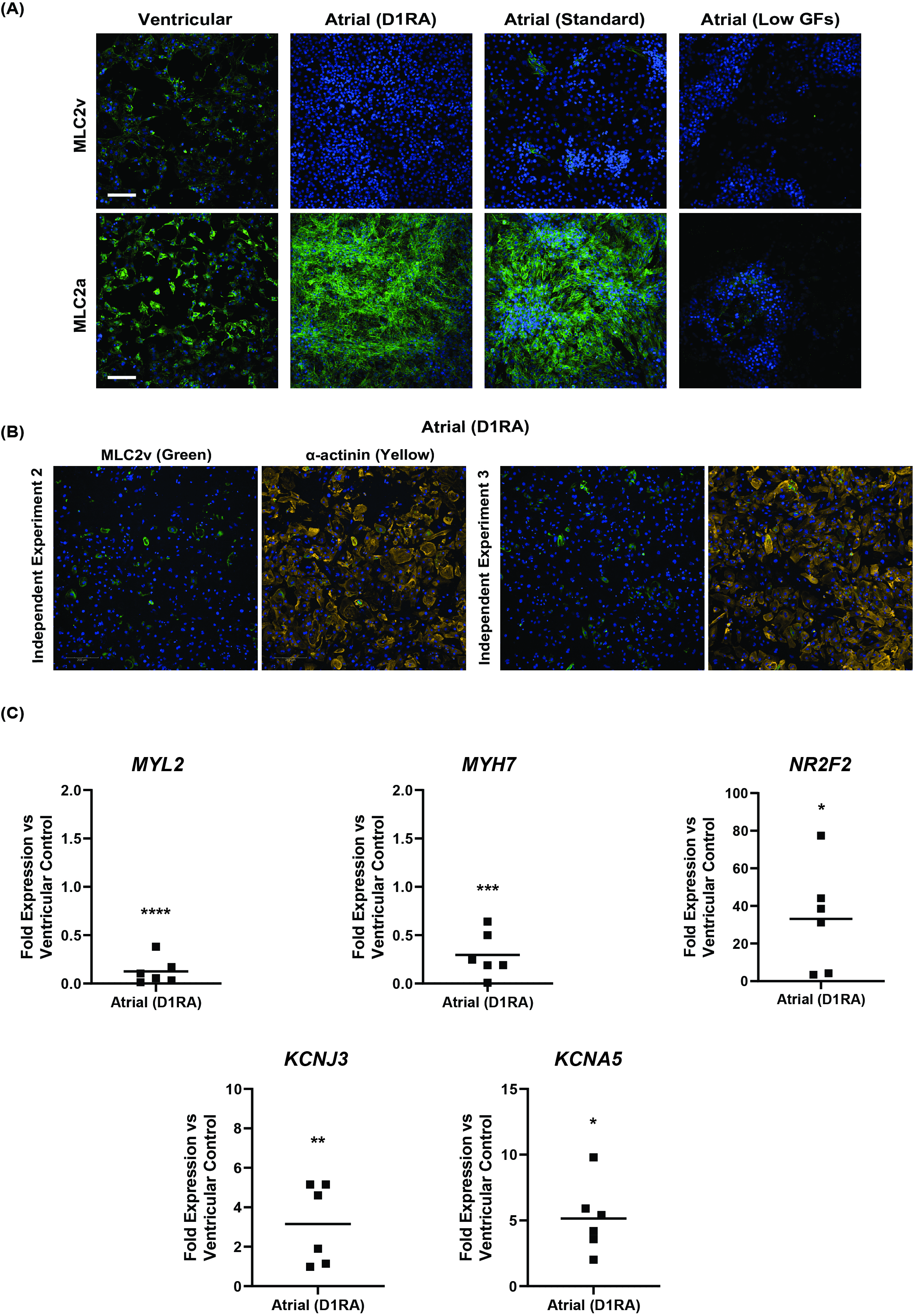

Supplement: Supplementary file 3 — Additional file 3: Fig. S2. Molecular characterization of atrial and ventricular differentiation methods. A To determine the effectiveness of the atrial differentiation method to produce atrial but not ventricular cardiomyocytes, cultures underwent immunostaining for ventricular marker (MLC2v) and atrial marker (MLC2a). Scale bars = 100 μm. B Immunostaining for MLC2v and α-actinin presence in two further independent experiments for Atrial (D1RA). C qPCR analysis of Atrial (D1RA) compared to matched ventricular controls. Unpaired t-test statistical testing performed for qPCR samples. [file 13287_2023_3405_MOESM3_ESM.jpg]

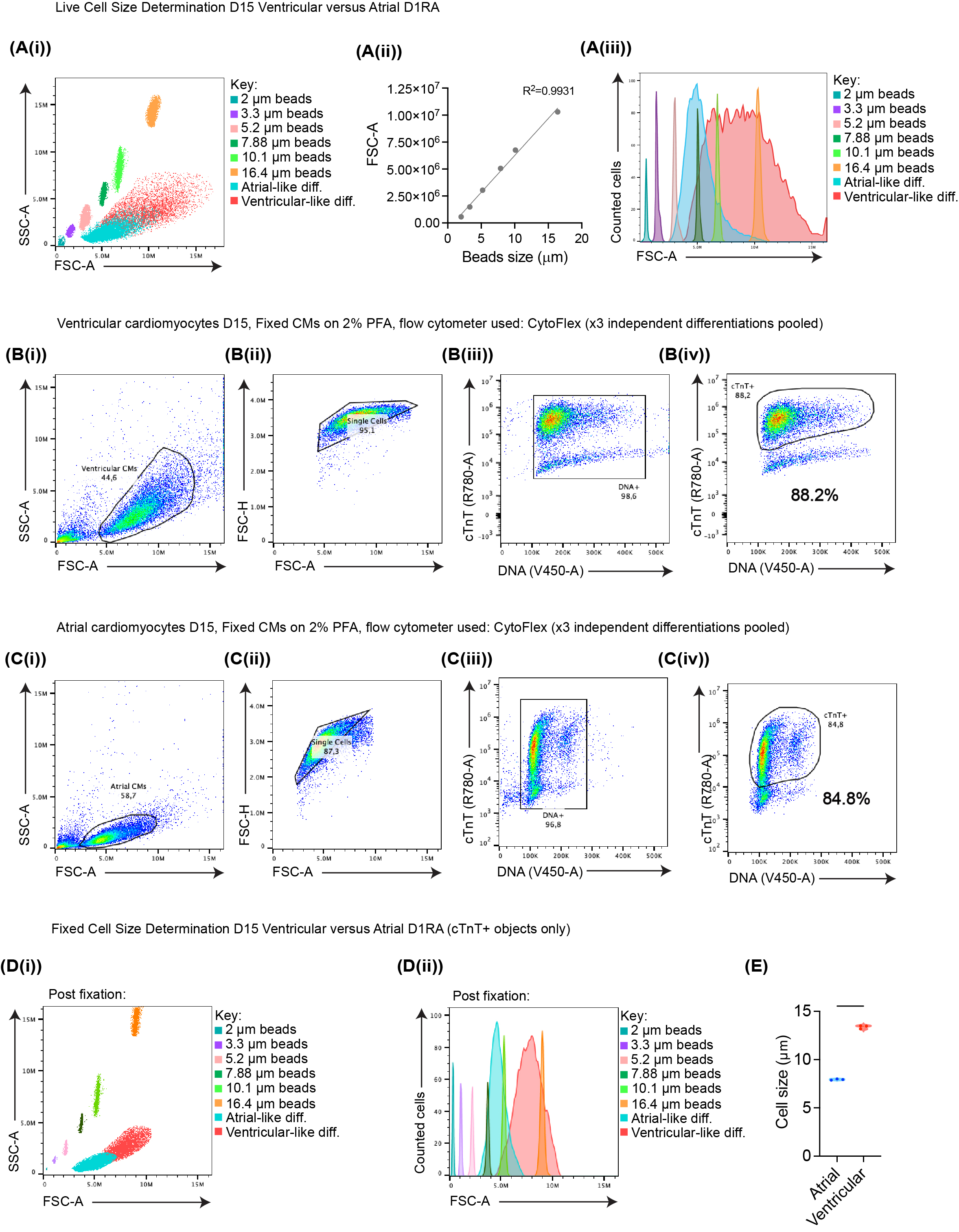

Supplement: Supplementary file 4 — Additional file 4: Fig. S3. Flow cytometry comparison of ventricular and atrial (D1RA) differentiation protocols. A Quantification of cell size from live populations, (i) comparison of forward and side scatter parameters of Atrial (D1RA) and ventricular protocol versus calibration beads of known size. (ii) Relationship between calibration bead size and forward scatter area parameter. (iii) Counted cells versus forward scatter. B and C Flow cytometry analysis for cTnT positive populations in ventricular and atrial (D1RA) protocols respectively, from three pooled independent experiments. D Quantification of cTnT+ populations for cell size from (B, C), against calibration beads of known size. [file 13287_2023_3405_MOESM4_ESM.jpg]
